# Social Workers’ Choice Making in Supporting Nature Activities by Parents and Children in Shelters

**DOI:** 10.3389/fpsyg.2022.891419

**Published:** 2022-06-15

**Authors:** Elise Peters, Dieuwke Hovinga, Jolanda Maas, Carlo Schuengel

**Affiliations:** ^1^Department of Education, University of Applied Sciences Leiden, Leiden, Netherlands; ^2^Department of Clinical, Neuro and Developmental Psychology, Faculty of Behavioural and Movement Sciences, Vrije Universiteit Amsterdam, Amsterdam, Netherlands; ^3^Section of Clinical Child and Family Studies, Vrije Universiteit Amsterdam,, Amsterdam, Netherlands; ^4^Amsterdam Public Health Research Institute, Vrije Universiteit Amsterdam,, Amsterdam, Netherlands

**Keywords:** integrating nature, shelters, recovery, building, secure base phenomenon, nature-based intervention

## Abstract

Visiting a natural environment such as a garden or park helps people to recover from stressful circumstances. Women’s shelters and homeless shelters have started to integrate nature in their work, especially for families who seek temporary refuge, with the aim to support parents’ functioning and resilience. For professionals who want to facilitate engagement with nature among their clients, it may be helpful to learn how other professionals choose nature activities for the support of parents. The current study was aimed to uncover how social workers choose a nature activity for the support of parents, resulting in a model that can be used as a reflective tool among shelter professionals. The model is based on an analysis of actions of professionals, captured in case descriptions written by shelter professionals about parenting supportive nature activities that they facilitated for families under their care. The model shows that social workers promoted a back-and-forth between children’s exploration away from the parent and being with the parent. In facilitating these interactions, social workers used nature as an environment with stress reducing and strengthening capacities for parents and as an environment with supportive qualities for children’s play. A dimensional framework was extracted that described how professionals may choose activities.

## Introduction

When families have no safe or suitable living place and have support needs that cannot be met in their informal network, shelters can provide temporary homes. Homeless shelters focus on people who have no suitable home, for example due to home eviction after financial problems, and women’s shelters focus on women who are unsafe due to threat and abuse. Shelters provide physical safety and shelters professionals offer psychological support, arrange work and finances, and help families obtain permanent independent housing, aiming for a return to independent family functioning (Council of Europe, 2011; Lisbon Declaration on the European Platform on Combatting Homelessness, 2021). Notwithstanding these efforts, shelter life can also bring stressors to parental functioning that can impede independent family life. Parents have reported noise and chaos in the shelter living spaces, imposed shelter rules that do not match with parents’ own rules and routines, experiences that reduced self-esteem in their parental role, feelings of distrust toward shelter service provides, challenges to parental mental health, lack of material resources for parenting, and issues with stigma and negative stereotypes of homeless parents, that bring challenges to maintaining parents’ wellbeing, household routines and family functioning (Pable, 2012; Glenn and Goodman, 2015; Anthony et al., 2018; Bradley et al., 2018; Sylvestre et al., 2018; Matthews, 2021; Vrabic et al., 2022). It is important that shelter organizations find ways to make shelter experiences beneficial or at least not adverse to parental functioning.

Visiting a natural environment such as a garden, children’s farm, a forest, or park can be supportive to families. Studies in shelters as well as in other living places have indicated that natural environments near a family’s living place can be used as a safe and engaging place for family activities, where parents can find fun and unconstrained ways to interact with their children (Ashbullby et al., 2013; Izenstark et al., 2016, 2021; Cameron-Faulkner et al., 2018; Millican et al., 2019; Kotozaki, 2020; Rantala and Puhakka, 2020; Peters et al., 2020a; Varning Poulsen et al., 2020). Such positive moments in nature are associated with stress reduction in parents (Razani et al., 2018; Kotozaki, 2020) and responsive interactions between parent and child (Cameron-Faulkner et al., 2018). For parents in shelters specifically, experiences in a natural environment have been associated with parents’ experiences of connectedness with their child, autonomy in making parenting decisions, and competence in their parenting practice (Peters et al., 2020a,b, 2021). These findings suggest that professionals may use engagement with nature to support parents in shelters.

Several shelters have integrated nature in their practice to support parents’ functioning and resilience (Renzetti et al., 2014; Lygum et al., 2019; Millican et al., 2019; Norton et al., 2020; Peters et al., 2020a,b; Varning Poulsen et al., 2020) such as by offering seasonal celebrations in nature, walk and talk therapy, outdoor adventure experiences, therapeutic horticulture, or outdoor play moments. Thus far, little is known about how professionals choose nature activities for the support of parents. If helping families to engage with nature is to be part of professional skills and training, description and understanding is needed of choices that professionals might implicitly or explicitly make, when determining whether a nature activity may be good for a family.

The current study was aimed to describe the choices that social workers make when they facilitate a nature intervention with the intention to support parents. Professionals’ choices can best be constructed on observations of the actual behavior of professionals, so that the choices can be inferred from their actions in practice (Ryle, 2009; Kinsella, 2010; Schön, 2017). To collect data on the actual behavior of professionals, case descriptions with detailed descriptions of actual actions of the professional and the behavior of their client can be informative (Argyris and Schon, 1974). In this study, we collected and analyzed case descriptions that shelter professionals wrote about parenting supportive nature activities that they facilitated for families under their care.

## Materials and Methods

### Participants

This study included 99 shelter professionals who worked in child and family social work in a shelter for homeless families or in women’s shelters in the Netherlands during the study period (October 2018 until February 2019). The shelter professionals were selected from 20 shelters that participated in a Dutch nationwide project aimed to enhance the wellbeing of families in shelters through the development and use of natural environments. One year prior to data collection, each shelter had received funding for the design and landscaping of natural places. Each shelter developed a restorative garden, a natural play area, a children’s farm, and/or a vegetable garden.

Shelter managers were asked to include team members for participation in this study on the basis of being a professionally educated child and family social worker, being motivated to use nature in shelter social work, and being motivated to participate in research. Shelter managers provided the researchers with a list of team members who fitted the inclusion criteria, after which researchers contacted the professionals to inform them about the goal of the study and their rights as participants. Participating professionals signed for informed consent. For the participant flow, see Table 1.

**Table 1:** Participant flow.

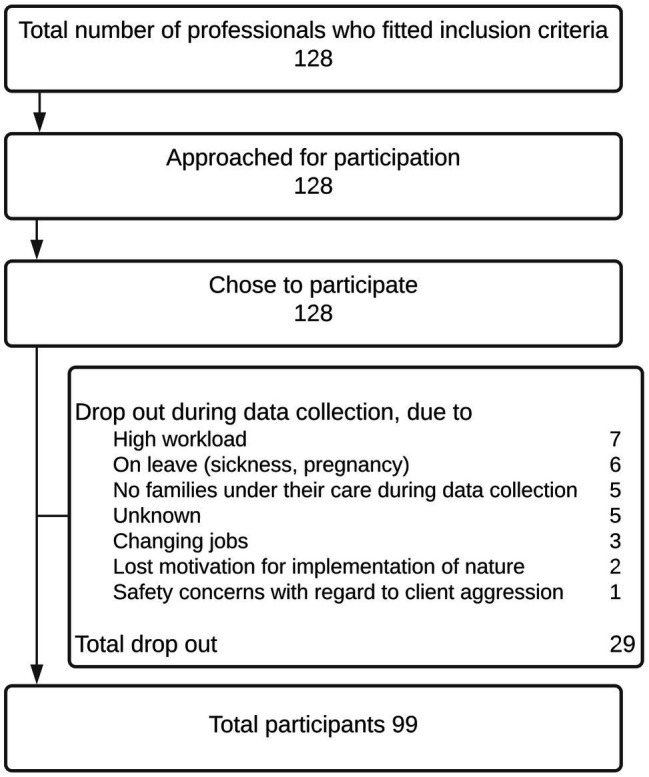

All participating professionals were educated in child and family social work in secondary vocational training, bachelor education, or master education. Their training includes assessing the needs of their clients by listening, questioning, observing, and professionally weighing these sources of information to form a professional judgment, as well as in facilitating activities in the support of their clients, and in critically reflecting on the effects. For participant characteristics, see Table 2. Professionals participated during their regular and paid working hours. Shelters could claim the expenses for professionals’ time spent on participation in the research, with a maximum of 32 h per professional at their hourly rate.

**Table 2:** Participant characteristics.

Gender	92 female, 7 male
Position	62 social worker/case manager/personal coach (educated in BA-education) 32 group worker/child and youth worker (educated in vocational education) 4 child and family counselor (educational level unknown) 1 family therapist (educated in MA-education)
Type of shelter	13 women’s shelter 5 shelters for homeless families 2 combined women’s shelter/ shelter for homeless families

All participating professionals took four training sessions in which they shared ideas for and experiences with nature activities for families under their care and reflected on their practice in conversations with colleagues, with the aim to develop, maintain, and share professional insights on nature activities for parents in shelters. After the second and third training session, data collection took place.

### Nature Activity Case Descriptions

Professionals facilitated a nature activity for a family under their care, either on the shelter property or in natural environments near the shelter. Nature activities were offered according to the family’s possibilities, preferences, and needs. Immediately after the nature activity, professionals wrote a case description with the date and time, the name of the shelter, a written observation of the parent’s parental needs based on the question “What needs did the parent have at this moment (based on your professional judgment)?”, a written description of the nature activity based on the question: “You facilitated a nature activity. What exactly happened? Describe the activity,” and a written observation based on the questions: “What did you notice in the parent? And what else? And what else?”. Parents filled out an online questionnaire about their parental need satisfaction and need frustration and their connectedness to nature, of which the results have been published (Peters et al., 2020b).

The Scientific and Ethical Review Board of the Faculty of Behavioral and Movement Sciences of the VU Amsterdam approved of the study protocol (VCWE-2018-0138).

### Analyses

Data consisted of 160 case descriptions. Eleven cases were removed because they had missing data or because the case described no nature activity with a family (for example, cases that described a professional with a child during a nature activity, without its parents). This resulted in a total of 149 cases for analysis. The data was thematically analysed using Atlas.ti 9.0.5 for Mac, using a Grounded Theory approach (Glaser and Strauss, 2017). The analysis was aimed to identify professional decisions in choosing nature activities for families in shelters One researcher conducted the analyses, supported by two researchers who parallel coded parts of the data. Researchers discussed the codes and importance of the codes until consensus was obtained. Memoing was used throughout the process of analysis. The analysis followed six steps:

#### Open Coding

A principal analyst used the *in vivo* coding setting to explore and code the content, aiming for a set of codes that represented each case. A peer analysts coded portions of these same data, after which the three analysts jointly discussed the codes and importance of the codes until consensus was obtained.

#### Axial Coding

The principal analyst and two peer analysts jointly analysed an initial set of 50 cases by comparing data within codes to explore patterns and exceptions in the data regarding that particular code. We asked ourselves what central themes could be used to describe these cases, resulting in concepts. All cases were subsequently analyzed by the principal analyst by asking if each case could be properly represented using the initial concepts, to identify if other concepts were needed, or if existing codes should be made better applicable. During coding, constant comparison was used to repeatedly check ideas against data in order to avoid confirmation bias (Boeije, 2002). This cycle of coding and comparing continued in an iterative, non-linear fashion until saturation was reached.

#### Selective Coding

By exploring connections and through further combining and summarizing, the principal analyst extracted core components and presented and discussed these with the peer analysts.

#### Negative Cases Analysis

The principal analyst marked aspects of the data that were in contrast with the apparent patterns in the data as negative cases. She refined and broadened her analysis until the codes covered almost all cases, which required the analyst to expand and revise her interpretation until a maximum number of cases could be explained, including those that were initially marked as negative cases or “outliers.” Any cases that did not fit the final model were documented in the final report to allow re-evaluation by others (Onwuegbuzie and Leech, 2007; Anney, 2014).

#### Network

The principal analyst worked to identify structures in the data set by linking codes, creating hierarchy, and visualizing the codes in a network. This process formed a model that described the choices that professionals made in facilitating nature activities for the support of parents in shelters. The model was presented to the two peer analysts as well as to all co-authors, supported by quotes from the original data set, for questioning and discussion.

#### Validation of the Results

To control the interpretations and consistency in meaning making the results were presented to a focus group of six participating professionals. We used peer debriefing by discussing the results of the study to a group of four researchers in the field of environmental psychology. The principal analyst who conducted the analyses took an academic course on grounded theory to improve reflexivity. In this article we illustrated the findings with raw data to present findings within their context.

## Results

### Descriptives

Case descriptions portrayed nature activities with parents (age *M* = 31.95, SD = 6.79) and their children (age *M* = 5.2, SD = 3.69). Nature activities were conducted with one child (*n* = 97), two children (*n* = 34), three children (*n* = 10), four children (*n* = 3), or five children (*n* = 3) and one parent (*n* = 143) or two parents (*n* = 4; missing data *n* = 2).

### Professional Decision in Choosing Nature Activities

The process of axial coding resulted in a series of codes. Supplementary Material 1 shows the codes and the number of occurrences of each code. The codes from axial coding were summarized and combined, resulting in three major themes.

#### Theme 1: Choosing Practical Dimensions When Facilitating Nature Activities

This theme includes eight dimensions according to which professionals chose a position when facilitating a nature activity for a family.

1. Physical Activity: professionals chose between a more sedentary and more physically active activity.
2. Familiarity: professionals chose between a more well-known and a newer experience.
3. Nature Interaction: professional chose between looking at nature and interacting with nature.
4. Proximity: professionals chose between staying close to and going further away from the shelter.
5. Location: professionals chose between a nature activity in an indoor space and a nature activity in an outdoor space.
6. Predictability: professionals chose between working with more predictable elements of nature and more unpredictable elements of nature.
7. Autonomy: professionals chose between an activity that was largely supported by the professional and more autonomous family time.
8. Openness of the Assignment: professionals chose between an activity with a more directive assignment and with a more open (or no) assignment.

Supplementary Material 2 provides illustrative quotes for every dimension.

In choosing the practical dimensions, professionals made personalized choices for each family. As an example, when professionals aimed the nature activity to help a mother in making contact with her child, one professional chose for a directive assignment (Quote respondent 1131: “The mother is holding back and insecure in making contact with daughter. The frame of the assignment helps her to show engagement”), while another professional chose for an open assignment (Quote respondent 1471: “The mother gets enthusiastic from everything she sees. Some things trigger memories, like blowing on a whistle from an acorn hat that we found on the ground. But she also talks about everything you can find in the wood to use. Her son seems focused on mom: he listens and is interested in everything. In mother I see serenity, relaxation, diversion. Other emotions. Focus on her child”).

#### Theme 2: Choosing to Use Opportunities of Nature to Facilitate a Specific Experience

This theme identifies opportunities of nature that professionals used to facilitate a specific experience, namely nature’s opportunities for children to explore, nature’s opportunities for stress reduction for parents, and nature’s strengthening opportunities for parents.

Exploration opportunities were related to free play, as is illustrated in this example (Quote respondent 0741: “I see a child who wants to explore and the imagination that she shows; for example, putting the leaves she collected in her coat pocket, trading leaves with others, putting the leaves inside the toys she brought to see if they came out”). Professionals mentioned nature as an interesting play environment (Quote respondent 1551: “Both the playground and simply a frozen puddle of water provide a challenge to do something for and with the children”), they mentioned children’s freedom in play behavior (Quote respondent 1151: “The children were excited, entered the bushes and shrubs without hesitation, searched under and on top, got dirty and laughed about it”), and they mentioned children’s involvement in play (Quote respondent 1561: “Both the children play, have attention, have fun. Mother enjoys it. Children have their focus, playing is their activity in that moment”).

Stress reducing opportunities for a parent were related to recovering from stressors, often indicated by feelings of relaxation, escaping daily stressors, and reducing feelings of anxiety and worries, as is illustrated in this example: (Quote respondent 1641: “Mother looks relaxed and lets the children do their own thing in the woods. They are free to run and walk and play with the dog. Mother says she finds it calming to be outside in nature. I get the impression she feels at ease there”).

Strengthening opportunities for a parent were related to positive experiences, creating social bonds, connecting to the family’s past, and building (new) family routines, as is illustrated in this example: [Quote respondent 1511: “Because they both love animals and the petting zoo was just around the corner of their home, they used to go there often. While here, they have not been there ever, not for the past 7 months. I went to the petting zoo together with mother and daughter (…). Because they liked it so much and they enjoyed the animals and the fresh air so much, mother wants to do this more often. It is their moment together; in this way they share their love for animals”].

#### Theme 3: Choosing to Facilitate a Specific Pattern of Interaction Between Parent and Child

This theme identifies the pattern of interaction that professionals facilitated between parent and child during the nature activity. Professionals facilitated a pattern in which children switched between exploring away from the parent and seeking proximity to the parent. While children explored, parents functioned as a secure base by being available, responsive to the child’s needs, and providing effective comfort.

Children exploring away from the parents is illustrated in this example: [Quote respondent 0191: “The children immediately ran around in the outdoor playground. The oldest (boy) jumped on the swing in the shape of a nest, the three middle ones (girls) climbed on the playset and the youngest immediately spotted the chalk”], and children seeking proximity to the parent is illustrated in this example: (Quote respondent 0771: “They love feeding the animals. Always asking for confirmation whether mother sees them when they give bread to one of the animals. Mother reacts positively”). Parents’ secure base behavior is shown in this example: [Quote respondent 1441: “When daughter comes over with a sad face (fallen) this changes by a single question from mother. Mother listens to what happened, and daughter soon says it’s better already”].

The pattern of children’s exploration and return while parents functioned as a secure base was present in almost all cases. This pattern of interaction was not always positive. In some cases, parents were not available (Quote respondent 0671: “Now and again the child sought out his mother to show what he had discovered or get her attention. Mother did not always know how to react, looked unsure of herself”), or restricted the child’s exploration [Quote respondent 1571: “Daughter often indicated she wanted to walk on her own, while mother was trying to hold her by the hand. (…) Mother felt the need to keep her daughter close to her. She seemed eager to control this. When I asked about it, she said this was correct, that she finds it difficult to let her daughter explore out there”].

### Negative Case Analysis

Two cases were in contrast with the rest of the data and were identified as negative cases. In these cases, professionals aimed for a different pattern for interaction between parent and child compared to the rest of the cases in the data set. Both negative cases described an activity in which professionals gave strict behavioral instructions to both parents and children with the intention to practice a new skill (psycho-physical resilience in one case, collaborative skills in the other case). In these cases, professionals left limited degrees of freedom to the child’s exploration. In the rest of the data professionals may also have described assignments and directions for behavior in parents in children, but still left room for initiatives from the parent or the child which allowed them to balance between proximity seeking and exploration.

### Network

**Figure 1:**
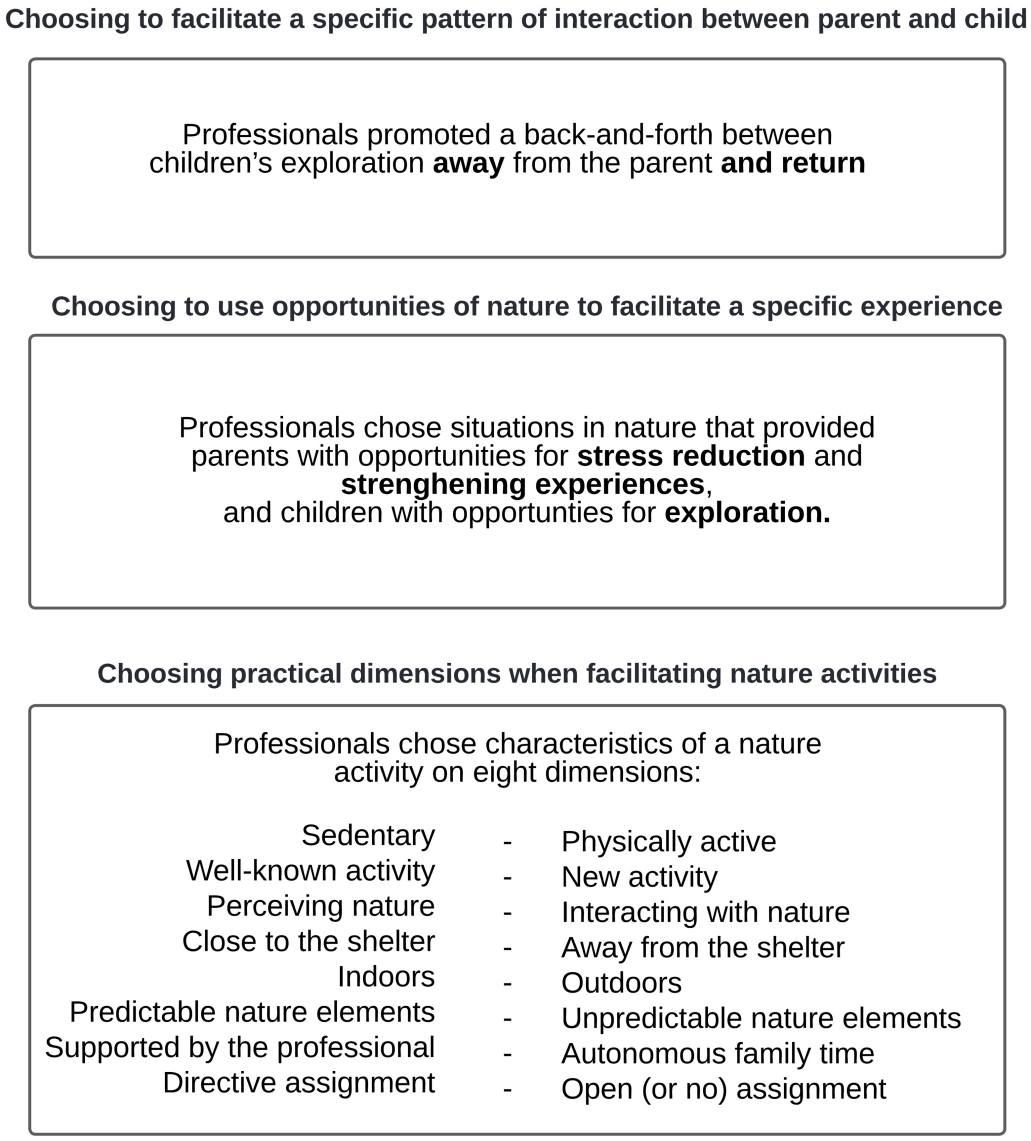
Professional choices in facilitating nature activities for the support of parents in shelters.

We made a network of codes to identify structures in the data set, with the aim to understand professionals’ choices when facilitating nature activities for the support of parents. The model presented in Figure 1 describes professionals’ choices as immerged from the data.

### Validation

For validation, results were discussed in a focus group with six professionals who had participated in the study, and with four researchers in the field of environmental psychology. The professionals recognized that they used nature as a setting with stress reducing and strengthening capacities for parents and exploration opportunities for children, and that they made choices on the eight dimensions to make the nature activity fitting for a specific family (Quote respondent 103: “I do indeed recognize that our social workers consciously think about relaxation moments for parents and play opportunities for children. As a team, we certainly think that nature is helpful in this”). All professionals in the focus group recognized that they chose activities that promoted a back-and-forth between children’s exploration away from the parent and return. Some professionals recognized their own role in this (Quote respondent 101: “I do try to sometimes involve a parent in the child’s activities: proximity; and sometimes stimulate a parent to let a child do something independently: distance. Sometimes you give some guidance, sometimes you just let things happen between parent and child”). Other professionals saw it as a side effect (Quote respondent 103: “I think that our family social workers are to a lesser extent consciously engaged in creating a balance in distance and proximity, but rather experience that this is a favorable side effect of working with nature,” Quote respondent 104: “At present the issue of distance/proximity and mutual contact between parent and child remains consciously observed by our colleagues but is not yet really pursued on purpose. It just happens, outside”). The professionals and the psychological researchers stressed that the choices for practical dimensions of the nature activities were partly influenced by practical considerations (Quote respondent 101: “But practical considerations, for example close to the shelter, free of charge, *etcetera*, are also decisive for the choice of activity”) and that decisions were not always planned prior to the activity but partially made in response to what happened during the activity.

## Discussion

With this study we aim to uncover shelter professionals’ choices when facilitating nature activities for the support of parents. In the current discussion we connect the results of the analysis to literature to demonstrate how the study results relate to extant knowledge within the field, using the jargon from the field (Stern, 2007).

### Linking Findings to Extant Literature

When facilitating nature activities for the support of parents in shelters, professionals chose to facilitate nature activities that promoted a back-and-forth between children’s exploration away from the parent, and return. These interactions followed the pattern of Secure Base Phenomenon (Ainsworth, 1967; Posada et al., 2013). Professionals facilitated such interactions, and only by exception opted for a different pattern for interaction (the “negative cases” in the Results). Other patterns may have occurred if professionals made different choices in the design of nature activities, for example by choosing activities that left little room for exploration, or by choosing activities in which parents could not be available for the child such as activities that caused high levels of arousal, or activities that required directed attention on something else than the child. Social workers’ primary focus on creating interactions according to secure base phenomenon is in line with a recent finding that child care professionals rely on Attachment Theory most often for their child supportive work (Department of Education, 2018).

In facilitating secure base interactions, professionals used nature’s capacities for supporting children’s exploration, and nature’s capacities for facilitating stress reduction and strengthening experiences. Regarding children’s exploration, professionals chose activities in which nature functioned as an interesting play environment that allowed children’s freedom in play behavior and stimulated their involvement in play activities. Theories on play suggest that natural environments can function as a setting for rich explorations (Nicholson, 1972; Heft, 1988). Natural environments are described as a setting that fits with children’s needs and desires for exploration and play (Spencer et al., 2019), where children play long, involved and diverse (Luchs and Fikus, 2013; Zamani and Moore, 2013). Professionals’ choices revealed that they set the scene for secure base behavior by choosing the environment so that it offered exploration opportunities for the child.

Regarding parents’ stress reduction and strengthening experiences, professionals chose activities in which nature offered opportunities for restoring energy, reducing feelings of anxiety, and experiencing positive interactions. Experts in the field of environmental studies have also described these capabilities of nature with the terms restoration and building (Markevych et al., 2017), with restoration referring to the ability of nature to restore resources that have been depleted in efforts to cope with stressors, and building referring to the deepening or strengthening of capabilities for meeting everyday demands (Marselle et al., 2021). Several theories were aimed to explain why natural environments can be experienced as non-threatening and stress reducing (Wilson, 1984; Kaplan and Kaplan, 1989; Ulrich et al., 1991; Kaplan, 1995; Kuo, 2015). Interestingly, recovery from stressors can been linked to higher psychological availability of parents, and more autonomy supportive and less controlling parenting behavior (Van Der Kaap-Deeder et al., 2019; Robichaud et al., 2020). Professionals’ choices revealed that professionals set the scene for secure base behavior by choosing the environment so that it facilitates parents to be available to their child and supportive of the child’s autonomy.

To facilitate exploration in children and stress reduction and strengthening experiences in parents, professionals made practical choices on eight dimensions. Professionals made unique choices on these dimensions for each family. According to Theory of Affordances (Gibson, 2014) every physical setting has unique properties for every individual. This means that, when aiming to facilitate certain behavior through engagement with an environment, one must consider the physical aspects of the environment as well as the characteristics of the family. Professionals’ choices show that they selected unique characteristics of a place to make its affordances fitting for a family.

### Notes for Interpretation

Professionals chose to facilitate interactions according to secure base phenomenon in their nature activities. This does not mean that we expect nature activities to be uniquely suitable for secure base interactions, nor that we expect professionals to use their knowledge on secure base phenomenon only during activities in nature. It may equally well be expected that professionals facilitate possibilities for secure base interactions on other moments in their professional practice, such as during indoor play moments or dinner time. This research shows that professionals also included secure base knowledge in their choices for facilitating nature activities.

The current study uncovers professionals integrated knowledge that is compatible with attachment theory in their choices for nature activities. Professionals might have well used other knowledge aspects, such as knowledge on physical fitness (which could have shown if professionals chose activities focused on building physical strength or getting vitamin D), or knowledge on social connectedness (which could have shown if professionals chose activities focused on connecting to the neighborhood or building friendships), or knowledge on self-connectedness (which could have shown if professional chose activities such as forest bathing, mindfulness, or yoga). It is of interest that professionals chose a social interaction perspective because it adds a new perspective to existing literature that has mainly focused on nature activities for physical health and mental wellbeing (Van den Bosch and Sang, 2017; Twohig-Bennett and Jones, 2018; Bratman et al., 2019; Lackey et al., 2021).

As professionals in the focus group highlighted, some aspects of the activity ‘occurred’ without professionals’ intentional guidance. Each activity reflected input from the professional, the parent, the child, and characteristics of the natural environment, that all interacted with each other, which makes the activity not only a result of the choices of professionals. The professional choices that were the focus of this study referred therefore to the input that professionals chose to provide in the decision process with the family about the nature activity.

### Strengths and Limitations

The study was conducted in the setting of a shelter, during regular working practice, with regular clients. This contributed to the ecological validity of the study findings. The study was conducted among a selected group of professionals. All professionals were educated in child and family social work, were selected by their team manager, worked in a shelter that had implemented nature to enhance the wellbeing of families, and were trained in the implementation of nature for parents. This allowed us to analyze data from professionals who we expected to be skilled and knowledgeable on the subject. Their choices were the basis on which a practice-based model was made.

No professionals dropped out during the informed consent procedure, but 23% of professional participants dropped out during data collection (see Table 1). Natural turnover of staff and clients, which is to be expected with a study period of 12 months, explained almost half of the dropout. The remaining dropout was 12%, which is acceptable (Furlan et al., 2009).

The data in this study consisted of case descriptions in which professionals described moments in their own practice. As Ryle (2009) argued, the mind of professionals is revealed in their doings, and explainable by the doers’ aims. This makes case descriptions suitable material for analyzing professional choices. We recognize three limitations in the way we collected case descriptions. Firstly, the case descriptions were limited in richness. Case descriptions were based on a predefined set of questions, which limited the options for rich elaborations by professionals, and limited opportunities for researchers to ask further questions. The written accounts by professionals were a representation of their actions in practice, but a first-hand involvement from the researchers, using their reflective and interpretive stance in interaction with the professionals, could have deepened our understanding. Secondly, professionals’ desired behavior, intentions, and world views could have interfered with their descriptions of their actual behavior. Professionals wrote about their own practice, which is already a reflection on their behavior (even though it was written immediately after the nature activity) and not necessarily an actual representation of the behavior itself. Professionals may have filtered their actual actions through the lens of their desired behavior, to make it sensible, logical, and concurrent with their values. A relational approach in data collection with researchers closer to the professionals, e.g., by actively observing professionals in action, could have strengthened the study. Thirdly, data collection and analysis were performed separately, which prevented theoretical sampling (Glaser and Strauss, 2017). Theoretical sampling could have made further examination of the categories and their relationships possible, such as an examination of the conditions under which certain characteristics of nature activities were chosen.

### Implications for Practice

This study describes the daily practical choices that professionals make when facilitating a nature intervention for a family in their care with the intention to support parents. The results of the study can be used by professionals who aim to implement nature to support parents in their practice. The results can make professionals aware of the choices that other professionals make. On the one hand, such insight may be interesting for professionals who are beginners in the use of nature activities in their parenting supportive practice. For them, the results can function as a “cheat sheet” on which they can see how more experienced colleagues make their choices, and so inform their own choices for facilitating nature engagement among their clients. On the other hand, the insights from this study may be used by professionals to reflect on practice. The results can function as a ‘mirror’ that reflects current choices. Such a ‘mirror’ can stimulate reflective conversation, for example by discussing if the choices reflect what professionals consider good practice. To aid this practical use of the study results, we made a printable poster that can function as a reflective tool for practice (Supplementary Material 3).

The data of this study have been collected on shelters that had some form of nature on their own property, safe and open to the families. Shelter professionals who do not have a natural environment at their disposal may be limited in the choices available to them. Especially for clients for whom safety is at risk or who face financial barriers, professionals will be very limited in choosing to go outdoors, to go further away from the shelter, or to facilitate autonomous family time in nature, if no safe and affordable natural environment of their own choice is available. It may well be that a safe garden on the shelter’s property is an important prerequisite for being able to make apt professional choices. If professionals are limited in making the choices from the model due to practical constraints, the model cannot function as intended. We invite professionals to make an analysis of the natural environments they have available at work, to determine whether using the model fits to their practice.

## Data Availability Statement

The raw data supporting the conclusions of this article will be made available by the authors, without undue reservation.

## Ethics Statement

The studies involving human participants were reviewed and approved by The Scientific and Ethical Review Board of the Faculty of Behavioral and Movement Sciences of the VU Amsterdam (VCWE-2018-0138). The patients/participants provided their written informed consent to participate in this study. Written informed consent was obtained from the individual(s) for the publication of any potentially identifiable images or data included in this article.

## Author Contributions

EP, DH, JM, and CS: conceptualization. EP and DH: funding acquisition. EP: data collection and writing—original draft. EP and JM: analysis and interpretation. DH, JM, and CS: supervision and writing—review and editing. All authors contributed to the article and approved the submitted version.

## Funding

The study is partly funded by Stichting Kinderpostzegels Nederland under grant “Huisje Boompje Beestje.”

## Conflict of Interest

The authors declare that the research was conducted in the absence of any commercial or financial relationships that could be construed as a potential conflict of interest.

## Publisher’s Note

All claims expressed in this article are solely those of the authors and do not necessarily represent those of their affiliated organizations, or those of the publisher, the editors and the reviewers. Any product that may be evaluated in this article, or claim that may be made by its manufacturer, is not guaranteed or endorsed by the publisher.
