# Supplementary material for: Social Workers’ Choice Making in Supporting Nature Activities by Parents and Children in Shelters
Source: Front Psychol. 2022 Jun 15;13:891419. doi: 10.3389/fpsyg.2022.891419 (PMC9240810; doi:10.3389/fpsyg.2022.891419)
Supplement: Supplementary file 1 [file Data_Sheet_1.pdf]

Appendix 1

Number of code occurrences in axial coding

Table 1a: Code occurrences for the practical dimensions of the nature activity

Codes	Number of occurrences in the data
A nature activity in an outdoor space	128
Supported by the professional	115
Physically active activity	88
Interacting with nature	86
With predictable elements of nature	75
Going away from the shelter	69
An open (or no) assignment	62
A directive assignment	44
With unpredictable elements of nature	37
Staying close to the shelter	15
Well-known activity	14
Looking at nature	14
New experience	10
Sedentary activity	10
Autonomous family time	8
A nature activity in an indoor space	6

Table 1b: Code occurrences for the type of experience that professional facilitated

Codes	Number of occurrences in the data
Creating opportunities for free play	62
Creating opportunities for relaxation	55
Creating opportunities for positive experiences	22
Creating opportunities for (re)building family routines	18
Creating opportunities for escaping daily stressors	12
Creating opportunities to connect with the family's past	11
Creating opportunities for restoring energy	11
Creating opportunities for reducing feelings of anxiety or worries	8
Creating opportunities for social bonds	8
Creating opportunities to be in the moment	7

Table 1c: Code occurrences for the type of interaction that professionals facilitated between parent and child

Codes	Number of occurrences in the data
Parent enjoying the child	124
Parent and child sharing experiences	117
Parent being available to the child	70
Child exploring away from the parent	70
Parent not responsive to the child	48
Parent supporting the child	47
Child seeking proximity to the parent	43
Parent guiding and correcting the child	39
Parent protecting the child	20
Parent comforting the child	9
Parent understanding the child	9
Parent being predictable for the child	6
