# Supplementary material for: Social Workers’ Choice Making in Supporting Nature Activities by Parents and Children in Shelters
Source: Front Psychol. 2022 Jun 15;13:891419. doi: 10.3389/fpsyg.2022.891419 (PMC9240810; doi:10.3389/fpsyg.2022.891419)
Supplement: Supplementary file 2 [file Data_Sheet_2.pdf]

Appendix 2

Eight practical dimensions between which professionals chose a position when facilitating a nature activity for a family: illustrative quotes

**Dimension 1:**<br>**Physical activity**	Professional's choice:<br>Sedentary	 Physically active
	Example from data, respondent 0091:<br>"Mother was mainly involved in observing, the children amused themselves especially with the leaves."<br>(Sedentary)	Example from data, respondent 0351:<br>"End of the afternoon ran outside together. The whole world was white! We started to throw snowballs to one another. Laughing and running! (…) A little later he also began to throw (snowballs) and to hide himself! (…) Afterwards worked together at making a large snowman."<br>(Physically active)
**Dimension 2:**<br>**Familiarity of experience**	Professional's choice:<br>New activity	 Well-known activity
	Example from data, respondent 0001:<br>"Mother is clearly not used to undertake activities outdoors with the children. She said she would sit herself down on the bench while the children play together. When I suggested it might be fun to play together, she was surprised. She first could not believe that she was to hide herself as well. Once she understood the game, she was clearly enjoying it."<br>(New activity)	Example from data, respondent 0051:<br>"Mother and daughter went out to play football. They regularly do this. You could tell they are well attuned to each other."<br>(Well-known activity)
**Dimension 3:**<br>**Nature interaction**	Professional's choice:<br>Perceiving nature	 Interacting with nature
	Example from data, respondent 0561<br>"I am going outside with mother and son to do some painting in nature. They are going to paint the surroundings and can choose the spot they want to paint themselves."<br>(Perceiving nature)	Example from data, respondent 0641<br>"Went out with mother and daughter to visit the mother of her friend. There we stroked a rabbit, we offered it food and drink, and cleaned the cage together with mum."<br>(Interacting with nature)
**Dimension 4:**<br>**Proximity**	Professional's choice:<br>Close to the shelter	 Away from the shelter
	Example from data, respondent 1171	Example from data , respondent 0501

	"Mother and children were not able to participate in the winter walk because of security issues. They cannot yet leave the premises. We organised a treasure hunt in the garden for them, with the kids searching for the hidden treasures."<br>(Close to the shelter)	"We cycled to the woods with a number of mothers and children. There we went for a walk."<br>(Away from the shelter)
**Dimension 5: Location**	Professional's choice:<br>Indoors	Outdoors
	Example from data, respondent 0621<br>"When we came in and saw the puppy dog a huge smile came to his face. We all sat down on the floor and first discussed what (not) to do with and near the doggy."<br>(Indoors)	Example from data, respondent 0111<br>"Madame went outside with her children (+personal counsellor). In the playground the children went out to play."<br>(Outdoors)
**Dimension 6: Predictability**	Professional's choice:<br>Focus on Unpredictable elements	Focus on Predictable elements of nature
	Example from data, respondent 0381<br>"Then we passed a park with animals, children both love and somewhat fear animals. A nice combination for both children. The eldest, age 5, is very afraid of dogs and the youngest, age 2, not at all. Mother stimulated the kids to stroke and feed the animals. This went well and gave lots of fun."<br>(Focusing on unpredictable elements of nature)	Example from data, respondent 0611<br>"Once in the park we sat on a bench for a while and enjoyed the surroundings and played 'I spy with my little eye'."<br>(Focusing on predictable elements of nature)
**Dimension 7: Autonomy**	Professional's choice:<br>Supported by professional	Autonomous family time
	Example from data, respondent 0991<br>"I demonstrated how to do the assignment. After doing it myself a few times I let mother take the initiative to do the activity with her child and I observed what happened."<br>(Supported by professional)	Example from data, respondent 0701<br>"The parents went to play in the snow with the children. The parents and the children attacked each other with snowballs. The children were chased and chased the parents."<br>(Autonomous family time)
**Dimension 8: Openness of the assignment**	Professional's choice:<br>Directive assignment	Open (or no) assignment
	Example from data, respondent 0611<br>"Then I explained the next assignment. Together all sorts of autumn leaves, twigs, feathers, and seeds/fruits were collected. These were collectively stored in one bag. On a clearing in the	Example from data, respondent 0331<br>"They played outside together, the eldest son did not fancy it very much but loosened up more and more while playing. They went on the seesaw,

	park, we played some ball games (throw a ball in the bucket, throwing and catching while counting).”<br>(Directive assignment)	went off the high slide together, chased each other, the atmosphere was relaxed.”<br>(Open (or no) assignment)
