# Supplementary figures and images for: Social Workers’ Choice Making in Supporting Nature Activities by Parents and Children in Shelters
Source: Front Psychol. 2022 Jun 15;13:891419. doi: 10.3389/fpsyg.2022.891419 (PMC9240810; doi:10.3389/fpsyg.2022.891419)

## Appendix 3

*A reflective tool for professionals*

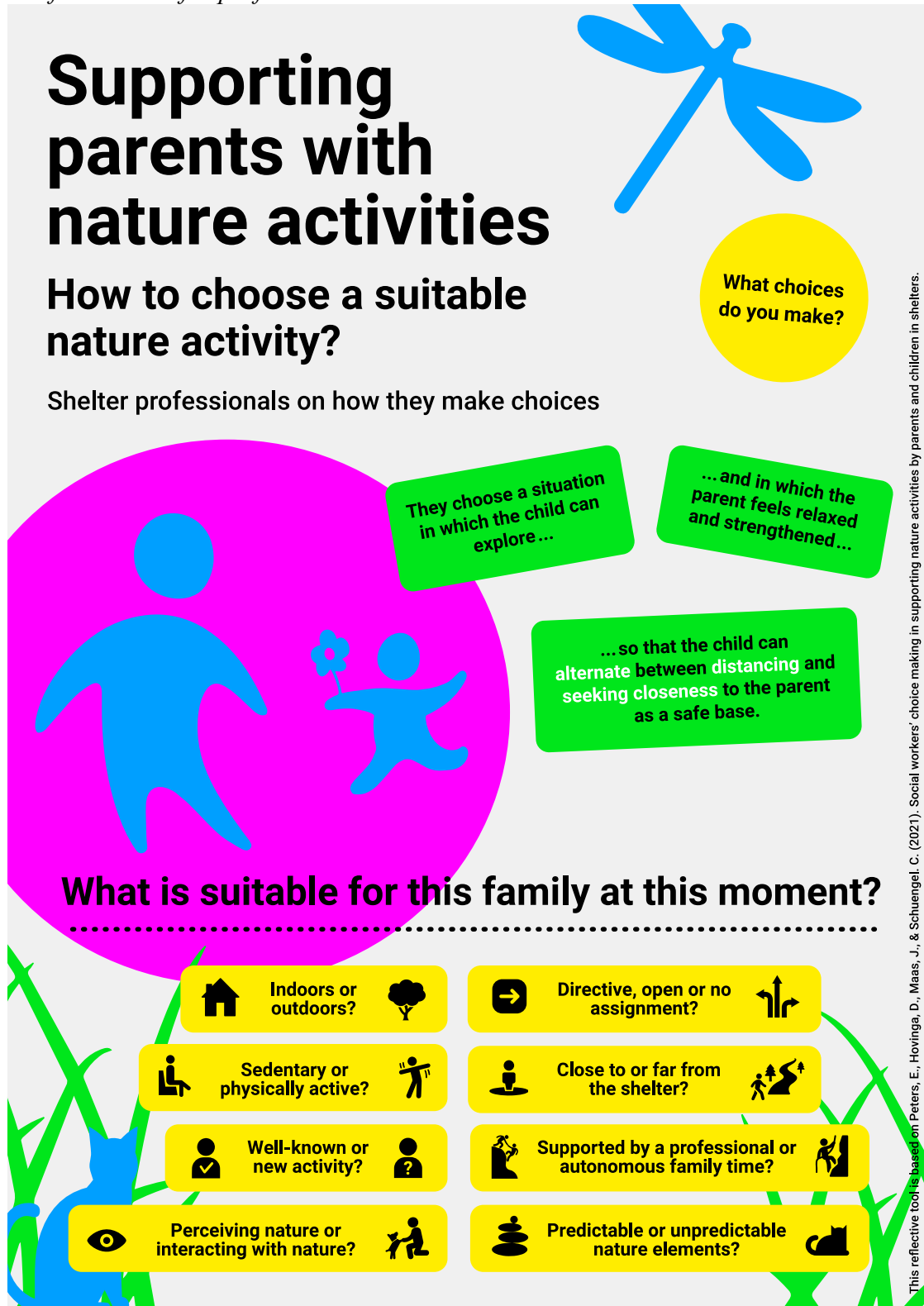

Supplement: Supplementary file 3 [file Data_Sheet_3.pdf]
